# The safety and efficacy of on-site paramedic and allied health treatment interventions targeting the reduction of emergency department visits by long-term care patients: systematic review protocol

**DOI:** 10.1186/s13643-018-0868-5

**Published:** 2018-11-23

**Authors:** Shannon Leduc, Peter Kelly, Venkatesh Thiruganasambandamoorthy, George Wells, Christian Vaillancourt

**Affiliations:** 1 Ottawa Paramedic Service, 2465 Don Reid Dr, Ottawa, Ontario K1H 1E2 Canada; 2 Department of Emergency Medicine, University of Ottawa at The Ottawa Hospital, 1053 Carling Ave, Ottawa, Ontario K1Y 4E9 Canada; 3 Clinical Epidemiology Unit, F6 Ottawa Hospital Research Institute at The Ottawa Hospital, 1053 Carling Ave, Ottawa, Ontario K1Y 4E9 Canada; 4 Cardiovascular Research Methods Centre, University of Ottawa Heart Institute, 40 Ruskin Street, Ottawa, Ontario K1Y 4W7 Canada

**Keywords:** Prehospital, Paramedic, Long-term care, Alternative care, Treat on-site, Treat and release, Community paramedic, Nursing home

## Abstract

**Background:**

Older adults are more likely to access the emergency department, which suffers from overcrowding and congestion, for conditions that could potentially be treated in other settings. Older adults living in long-term care centers have access to healthcare resources in their residence, and several programs have been created with the intent of treating medical conditions on-site. The aim of this study is to identify and systematically review programs and interventions at long-term care centers that aim to treat patients on-site, avoiding unscheduled transportation to the emergency department.

**Methods:**

We will follow the Preferred Reporting Items for Systematic Reviews and Meta-analyses (PRISMA) guidelines. We will perform a comprehensive search of Embase, MEDLINE, CINAHL, ClinicalTrials.gov, PROSPERO, and the Cochrane Central Registry of Controlled Trials using a broad search strategy. Two independent reviewers will assess titles and abstracts against inclusion criteria, and we will further evaluate relevant full-text articles for inclusion. We will assess the risk of bias using the Newcastle-Ottawa scale for included non-randomized studies and the Cochrane Risk of Bias tool for randomized trials. We will present a narrative synthesis of results and complete a meta-analysis only if enough homogeneity is found. We will create funnel plots to evaluate possible reporting bias and use The Grading of Recommendations Assessment Development and Evaluation (GRADE) methodology to assess the confidence in cumulative evidence.

**Discussion:**

As pressure on the healthcare system continues to rise, many areas are looking for alternative models of care. Several programs have been put in place in long-term care centers that seek to avoid transportation to the emergency department by providing enhanced care on-site. These programs are quite variable, and, to date, there is no standardized program or model of care.

**Systematic review registration:**

PROSPERO (CRD42018091636)

## Background

The population of Canada is aging, living longer, and utilizing more healthcare resources than previous generations [1]. These factors add to the challenges faced by an already overwhelmed healthcare system that is struggling to cope with high demand and limited resources. In particular, emergency departments (ED) are crowded, resulting in long waits for care, and negative patient outcomes [2, 3]. This congestion has a ripple effect which impacts paramedic services and patients waiting for emergency care in the community. Emergency department congestion contributes to long waits for paramedics to transfer the care of their patients to the ED staff, referred to as offload delay, which in turn delays paramedic response to the next emergency call. At times, offload delays have put so much pressure on emergency services that there have been no ambulances available in the community to respond to the next emergency call [4–9]. It is in this context that different factions of the healthcare community have been investigating alternative models of patient care to relieve the pressure on paramedics, emergency departments, and the healthcare system.

Older adults use hospital services more than other age groups and are more likely to access the emergency department for conditions that could potentially be treated in other settings [1]. Although only a small proportion of emergency visits are from long-term care patients, this group is more vulnerable to adverse events from the transfer of their care to the emergency department [10]. They also have access to health care resources in the long-term care center, making them a group of interest for interventions that provide care in the place of residence avoiding ED visits. Community Paramedic programs, where paramedics augment the care of individuals in the community, are one example of these programs. Several such programs have been put in place with models of care that can vary significantly from program to program [11–15]. There has been no systematic evaluation of these programs to date.

The aim of this systematic review is to identify programs and interventions that avoid transporting long-term care patients to the emergency department by providing care on-site and to evaluate the safety and efficacy of these programs.

## Methods

### General methods

We will complete a systematic review and report a narrative synthesis. We will conduct a meta-analysis only if appropriate. This systematic review is registered with the international prospective register of systematic reviews PROSPERO (CRD42018091636). We have adhered to the Preferred Reporting Items for Systematic Review and Meta-Analysis Protocols (PRISMA-P) 2015 statement and the completed checklist is included as an additional file [16].

### Objective

Specifically, the systematic review will answer the following questions:

1. Among patients living in long-term care facilities, what is the safety and efficacy of interventions that treat patients on-site, avoiding unscheduled transportation to the emergency department?
2. Among patients living in long-term care facilities, what are the additional patient and system impacts of implementing treat on-site interventions beyond safety and efficacy?

### Eligibility criteria

We will use the following inclusion criteria to select studies for this systematic review:

- P—Patients living in long-term care facilities, which we define below.
- I—Any intervention or program provided by a non-physician that aims to reduce unscheduled patient transports to the emergency department
- C—Standard care or transportation to the emergency department
- O—All reported patient and system effects of any kind

### Participants

We will include studies that contain patients 18 years of age and older, living in long-term care facilities primarily meant for the elderly. These facilities must be long-term care and not retirement residences or assisted living facilities, as the medical care available to residents is different between these facilities as are the patterns of ED use. We will include a long-term care facility also caring for younger patients with chronic disease in addition to the main population of older adults, providing they are not a facility that specializes in the care of these younger adults. We will include studies that encompass any patient condition. We will exclude hospitals that specialize in chronic care.

### Interventions

We will include any intervention or program aimed at reducing the transport of patients to the emergency department. Programs where reducing transportation to the emergency department is a secondary goal will be considered, if the number of ED transports is measured. These programs can be preventative or for acute conditions. The intervention or program must have been completed by a care provider other than a physician, for example, a paramedic or a nurse. We may consider for inclusion programs where physicians are part of a multi-disciplinary care team on an individual basis, providing allied health providers play a significant role in the intervention. We will include programs or interventions for any conditions.

### Comparators

Usual care or transportation to the emergency department will be the comparator.

### Outcomes

We will collect outcomes taking a broad perspective. Outcomes will be collected as they are reported, and surrogate outcomes will be included if appropriate. In the event, outcomes are reported as composite measures, we will extract the composite and individual outcomes. Pre-defined outcomes of interest include the following:

1. *Patient outcomes:* the proportion of hospital admission, length of stay, death, condition-specific morbidity or adverse events, health-related quality of life, frailty measures, functional status, and patient impacts and perspectives. Quality of life, frailty measures, and functional status will only be collected if a validated tool was used.
2. *System outcomes:* the proportion or number of ED transports averted, repeat 911 or ED access, healthcare resource utilization, paramedic system response times, offload delays and ED crowding or congestion measures, and measures of overall system function (for example, end of shift overtime rates). We will consider other outcomes depending on what is available in the literature.

### Study designs

Study designs will be included taking a broad perspective. Experimental, quasi-experimental, and observational study designs will be included. We will exclude case reports, case series, and review articles, although we intend to review the reference list of review articles if any are found. Abstracts will be excluded unless a full-text article can be found.

### Timing

There will be no restriction based on length of patient follow-up time.

### Language

Articles reported in the English or French language will be included. If articles are found that appear relevant but full text is not available in English or French, we will include them in the Appendix.

### Exclusion criteria

We will exclude studies that relate to patients who live in dwellings other than long-term care centers and involve an intervention that is primarily led by a physician or does not have a significant involvement by allied healthcare providers and does not have the goal of caring for patients in place or reducing unscheduled transports to the emergency department.

### Information sources

We will search Excerpta Medica Database (Embase), MEDLINE, Cumulative Index to Nursing and Allied Health Literature (CINAHL), ClinicalTrials.gov, PROSPERO, and the Cochrane Central Registry of Controlled Trials with no date restrictions (SL). The reference lists of included studies, as well as any systematic reviews found, will be searched for other relevant articles (SL). We will search the primary authors of included articles to ensure all related research has been found. Gray literature will be searched using the pertinent areas of the Canadian Agency for Drugs and Technologies in Health (CADTH) Gray Matters Checklist (SL) [17]. We will present a list of included articles to subject matter experts and ask them to identify any additional documents that are not on the list. If clarification is required regarding study details, two attempts will be made to contact study authors. These attempts will be made 3 weeks apart.

### Search strategy

Medical subject headings and text words related to long-term care and hospital transfers have been used to create a search strategy. Our search strategy has been reviewed by Lindsey Sikora, Health Sciences Librarian at the University of Ottawa. A MEDLINE search strategy is included in the Appendix. We will adapt this strategy for Embase and CINAHL and test it to see if it yields a high proportion of known studies.

### Citation management

We will use Endnote software (version X7.5.3) to import references and abstracts from electronic databases. We will then use Covidence software to remove duplicates and facilitate the screening and tracking of articles. Full-text articles will be uploaded to Covidence for second-stage screening, and we will document the reason for exclusion for any articles excluded at this stage.

### Selection process

Stage 1—two independent trained reviewers will assess the title and abstract of each article identified by our search strategy (SL and second reviewer to be recruited). Articles thought to be relevant by both reviewers will proceed to stage 2, and we will report a measure of agreement. Stage 2—we will assess the full text of articles thought to be potentially relevant by both trained reviewers in stage 1, against the inclusion and exclusion criteria. Disagreements about inclusion decisions at either stage will be resolved by discussion and, if consensus cannot be reached, a third senior co-investigator will adjudicate (CV). If multiple articles are found that appear to be about the same work, we will assess these publications by examining author names, study locations, and intervention details to determine if they are duplicates.

### Data collection

One reviewer will collect data from included studies using a piloted standardized form (SL). If multiple articles are found that refer to the same study, we will link articles together. Data from each linked study may be collected onto its own extraction form and later combined, or one extraction form may be used for all linked studies depending on what types of duplicate publications are found.

### Data items

We will extract data on patient characteristics (age, gender, level of dependence, cognitive impairment), system characteristics (healthcare system payer information, type of prehospital system, levels of care providers in prehospital system), intervention characteristics (presenting patient condition, severity of illness, intervention details), long-term care center characteristics (number of beds, location, skilled care services, funding model), and study characteristics (study designs, methodologies and outcomes, funding). If long-term care center characteristics are not reported in the article but are publicly available, we will collect them.

### Outcomes and prioritization

1. The primary outcome will focus on the proportion of ED transports averted, defined as those patients who were treated at the long-term care center within the respective program, but not subsequently conveyed to the emergency department unless the transport to the emergency department was a scheduled visit. If patients were transported to an alternative destination, such as a clinic or family physician, we will collect that information but still consider it an avoided ED transport.

### Secondary outcomes included

2. The safety of interventions in long-term care homes and will include the proportion of patients who suffer adverse events. Adverse events will include return visits to the ED, hospital admission, death, or condition-specific adverse events. Given the potential for a broad range of patient conditions in the literature, we will not restrict the length of follow-up for adverse events that are reported as being associated with the patient’s episode of illness or treatment. Hospital admission, death, and condition-specific adverse events will be analyzed together as well as individually.
3. Hospital length of stay
4. Health-related quality of life as measured by general or disease-specific validated instruments
5. Frailty measures as measured by the Clinical Frailty Scale or another validated scale
6. Functional status as measured by a validated scale that is assessed by interview or observation but not self-reported measures
7. Patient perspectives as reported
8. The number of repeat 911 calls or emergency department access while under the care of the respective program, or within 7 days of completing the treatment program
9. Paramedic system response times, defined as the number of minutes it takes a paramedic or emergency medical worker to arrive on the scene of an emergency from the time the call for assistance is placed
10. The mean time in minutes that paramedics spend in offload delay, defined as the time waiting to transfer a patient to a hospital bed and hand over patient care after giving a report to the hospital staff
11. Measures of emergency department crowding or congestion as reported in the literature
12. Measures of overall emergency system function as reported. Examples may include the number of times a paramedic service has no available resource for the next emergency call (level zero) or are at critical levels of responder availability, end of shift overtime, response time standard compliance or the number of offload nursing hours required in the emergency department

The primary outcome will remain the same irrespective of the significance found in the included studies, or whether it has been reported as a priority in the literature. The definitions of individual outcome measurements may be altered depending on what is found in the literature. For example, if offload delay is more commonly reported in a measure other than mean time, the definition may be altered to reflect this. If there is an outcome reported in the literature that we have not considered and find important, we will collect it and state in the manuscript that it was not part of our initial list of outcomes. If standard errors are not reported in included studies, we will reconstruct them from confidence intervals and point estimates.

### Risk of bias

We will evaluate all included studies for risk of bias using a tool appropriate to study type. Randomized control trials will be assessed using The Cochrane Risk of Bias tool [18]. Risk of bias will be identified as low, high, or unclear in a table format for each domain. Each section in the Risk of Bias tool will be considered independently, and there will be no overall score calculated. If the risk of bias cannot be determined in a specific category because there is not enough information, the risk of bias in that category will be designated as unclear. The Cochrane Handbook instructions will be used to make a judgment on low- and high-risk bias ratings [19]. Non-randomized studies will be assessed using the Newcastle-Ottawa Scale [20]. The strengths and limitations of each study as well as the potential impacts they may have had on the outcome will be presented. Risk of bias will be determined independently by two reviewers and then compared for agreement. We will resolve any disagreement by discussion or third party arbitration if required.

### Data synthesis

We will summarize the characteristics and findings of all included studies, regardless of the risk of bias, in a narrative synthesis. We will present the results in table form as well as text. Any relevant subgroups that are discovered will be reported, and, when appropriate, similarities and differences between studies will be examined.

If included studies or a subgroup of included studies are homogeneous in patient conditions and study design, they will be meta-analyzed using a random or fixed effects model as appropriate. Studies will be assessed by two reviewers to determine if enough methodological and clinical homogeneity exists to warrant a meta-analysis. Dichotomous outcomes will be determined using odds ratios (OR’s) and 95% confidence intervals. Standardized mean differences with 95% confidence intervals will be used to analyze continuous outcomes. The standardized mean differences will be weighted by the precision of the estimates of each study. Study characteristics will be examined using descriptive characteristics. In the event of small sample sizes or skewed data, we will use non-parametric tests for analysis or the data will be presented descriptively. We will examine forest plots for statistical heterogeneity and factor in clinical relevance. The *I*^2^ statistic will also be considered with a value of 40% or greater considered moderate heterogeneity. A sensitivity analysis or subgroup analysis will be undertaken if heterogeneity is visible on the forest plots. Risk of bias, type of healthcare system, and funding models of the long-term care centers are potential variables for subgroup analysis. Subgroup analysis will also be completed for young patients with chronic diseases providing the study reports this group separately.

### Meta-bias assessment

To assess reporting bias, we will compare published protocols and trial registries with publications. We will evaluate if selective outcome reporting has occurred by comparing the outcomes that were planned and those that were published to see if there are differences. If greater than 10 studies are included in the review, funnel plots will be used to examine possible reporting bias.

### Confidence in cumulative evidence

We will use The GRADE methodology to assess the quality of evidence and will report the results in an evidence table [21–25]. Quality of evidence will be assessed independently for each patient outcome and will be rated as very low, low, moderate, or high according to the GRADE scale.

## Appendix

### Search strategy MEDLINE

1. Long-Term Care/
2. Homes for the Aged/
3. Nursing Homes/
4. Emergency Service, Hospital/
5. Emergency Medical Services/
6. (nursing adj2 home).tw
7. 1 or 2 or 3 or 6
8. on-site treatment*.tw
9. (emergenc* adj3 (respond* or aid* or technician*)).tw
10. (emergenc* adj3 service*).tw
11. paramed*.tw
12. ambula*.tw
13. Emergency Medical Services/ or “Transportation of Patients”/
14. exp “Transportation of Patients”/
15. (hospital adj2 transfer).tw
16. (patient adj2 trans*).tw
17. Allied Health Personnel/
18. Hospitalization/
19. 4 or 5 or 8 or 9 or 10 or 11 or 12 or 13 or 14 or 15 or 16 or 17 or 18
20. 7 and 19
21. limit 20 to “all adult (19 plus years)”

## Abbreviations

CADTH: Canadian Agency for Drugs and Technologies in Health
CINAHL: Cumulative Index to Nursing and Allied Health Literature
ED: Emergency department
EMBASE: Excerpta Medica Database
GRADE: Grading of Recommendations Assessment Development and Evaluation
OR’s: Odds ratios
PRISMA-P: Preferred Reporting Items for Systematic Review and Meta-Analysis Protocols
QoL: Quality of life
